# A Monoclinic V_1-x-y_Ti_x_Ru_y_O_2_ Thin Film with Enhanced Thermal-Sensitive Performance

**DOI:** 10.1186/s11671-020-03322-z

**Published:** 2020-04-22

**Authors:** Yatao Li, Deen Gu, Shiyang Xu, Xin Zhou, Kai Yuan, Yadong Jiang

**Affiliations:** School of Optoelectronic Science and Engineering, University of Electronic Science and Technology of China, Chengdu, 610054 Sichuan People’s Republic of China

**Keywords:** Vanadium oxide, Thin films, Titanium, Ruthenium, Thermal-sensitive

## Abstract

Preparing the thermal-sensitive thin films with high temperature coefficient of resistance (TCR) and low resistivity by a highly compatible process is favorable for increasing the sensitivity of microbolometers with small pixels. Here, we report an effective and process-compatible approach for preparing V_1-x-y_Ti_x_Ru_y_O_2_ thermal-sensitive thin films with monoclinic structure, high TCR, and low resistivity through a reactive sputtering process followed by annealing in oxygen atmosphere at 400 °C. X-ray photoelectron spectroscopy demonstrates that Ti^4+^ and Ru^4+^ ions are combined into VO_2_. X-ray diffraction, Raman spectroscopy, and transmission electron microscopy reveal that V_1-x-y_Ti_x_Ru_y_O_2_ thin films have a monoclinic lattice structure as undoped VO_2_. But V_1-x-y_Ti_x_Ru_y_O_2_ thin films exhibit no-SMT feature from room temperature (RT) to 106 °C due to the pinning effect of high-concentration Ti in monoclinic lattice. Moreover, RT resistivity of the V_0.8163_Ti_0.165_Ru_0.0187_O_2_ thin film is only one-eighth of undoped VO_2_ thin film, and its TCR is as high as 3.47%/°C.

## Introduction

Microbolometers have been widely applied in civil and military fields. One of the important development trends is reducing the pixel size in order to reduce product cost and increase the detection range [1]. However, the miniaturization causes the decrease of sensitivity. Improving the micro-electromechanical system (MEMS) manufacturing process to optimize the filling factor, absorption coefficient, thermal conductivity, and other key factors can effectively enhance the sensitivity, but this approach is coming to its limit [1]. Another effective way is using better thermal-sensitive materials [2]. As a widely used thermal-sensitive material, VO_x_ with a relatively low resistivity in the range of 0.1–5.0 Ω·cm has a TCR of about 2%/°C at room temperature [3]. Considering that the sensitivity of a microbolometer is proportional to the TCR, it is more favorable to use thermal-sensitive materials with higher TCR for increasing the sensitivity of small pixel microbolometers. In order to increase the TCR of VO_x_ films, Jin et al. prepared Mo-doped VO_x_ thin films by bias target ion beam deposition [3]. The films have a high TCR of − 4.5%/°C, but large resistivity (> 1000 Ω·cm) is not preferable for microbolometer applications.

For fabricating a typical VO_x_-based bolometer array, it is necessary to cover VO_x_ thermal-sensitive thin film with a passivation layer (SiN_x_ or SiO_x_), which can protect the thermal-sensitive thin film from the oxidation by subsequent processes (removing of photoresist, release of sacrificial layer, etc.) [4]. The protection effect of the passivation layer depends on its film density. Denser passivation layer results in better protection effect. Generally, high preparation temperature contributes to denser passivation layer [5, 6], thus better protection effect for VO_x_ thin films. However, VO_x_ thermal-sensitive thin films, which are generally prepared at relatively low temperature (lower than 300 °C), are amorphous [3, 7, 8]. Whereas amorphous VO_x_ tends to crystallize at elevated temperature [9]. Once the crystallization happens, electrical parameters of the film will be significantly changed. Therefore, relatively low preparation temperature for VO_x_ thermal-sensitive thin films constrains the process for the passivation protection layer. This causes an annoying problem for fabricating bolometer arrays: the very stringent control on the subsequent processes.

Monoclinic vanadium dioxide (VO_2_) thin films have been considered as a potential thermal-sensitive material for highly sensitive microbolometers owing to high TCR at room temperature (RT). Moreover, monoclinic VO_2_ thin films are prepared at higher temperature than 300 °C [10], which is beneficial for preparing denser passivation protection layer at higher temperature. However, the two characteristics of monoclinic VO_2_ limit, to a certain extent, its practical application for microbolometers. On the one hand, the semiconductor-to-metal transition (SMT) happens to VO_2_ near about 68 °C. The hysteretic feature and strain changes during the SMT of VO_2_ will deteriorate the device performance and reduce the reliability of the device [11]. On the other hand, relatively high RT resistivity (> 10 Ω·cm) restricts the choice of device operating parameters [12, 13]. Therefore, preparing the vanadium dioxide films with high TCR, non-SMT, low resistivity, and crystallization structure becomes a challenge for developing high-performance thermal-sensitive materials for microbolometers. Recently, Soltani et al. introduced both Ti and W into VO_2_ thin films in order to suppress the SMT [14], and prepared Ti-W co-doped VO_2_ thin films with non-SMT feature and a high TCR. However, Ti-W co-doped VO_2_ thin films have a similar resistivity to undoped VO_2_.

In this article, we demonstrate a high-performance monoclinic V_1-x-y_Ti_x_Ru_y_O_2_ thermal-sensitive thin film through a SMT-inhibition strategy by means of introducing Ti and Ru ions into VO_2_ thin films. The thin films were prepared by a reactive sputtering process followed by annealing at 400 °C. Higher process temperature than amorphous VO_x_ thin films provides more parameter choice of subsequent MEMS processes for bolometer devices. V_1-x-y_Ti_x_Ru_y_O_2_ thin films have similar monoclinic structure to undoped VO_2_, but the SMT feature is completely suppressed due to the pinning effect of high-concentration dopants. The thin film with optimal dopant concentration has higher TCR (3.47%/°C) than the commercial VO_x_ thin films, and much lower RT resistivity than undoped monoclinic VO_2_ thin films.

## Material and Methods

All the thin films were prepared through direct current (DC) reactive magnetron sputtering on quartz substrates (23 mm × 23 mm × 1 mm). A high-purity vanadium target (99.99%) with a diameter of 80 mm and a thickness of 4 mm was used for depositing thin films with a target-substrate distance of about 11.5 cm. After the base pressure is below 2.0 × 10^−3^ Pa, the sputtering was executed at 0.32A with an O_2_/Ar ratio of 1:50. During deposition, the substrate temperature was kept at 100 °C. Then as-deposited thin films were in situ annealed for 60 min at 400 °C in pure oxygen (4.4 sccm). The thickness of films was controlled as about 380 nm according to the calibrated deposition rate. Ti and Ru were introduced with pure Ti pieces (99.9% purity, 10 mm × 10 mm × 2 mm) and V/Ru alloy pieces (consisting of 10.0 at.% Ru and 90.0 at.% V, 10 mm × 10 mm × 2 mm) placed symmetrically on the sputtered surface of the V target. V_1-x-y_Ti_x_Ru_y_O_2_ thin films using 3 Ti pieces and 1, 2, 3 V/Ru alloy piece(s), Ti-doped thin film using 3 Ti pieces, and undoped VO_2_ thin film are marked as VTRO-1, VTRO-2, VTRO-3, VTO, VO, respectively.

The chemical states of dopants (Ti and Ru) were analyzed by X-ray photoelectron spectroscopy (XPS) with Al Kα radiation (1486.6 eV) using a ESCALAB 250 (Thermo instrument). The binding energies (BEs) were calibrated to the C 1 s peak at 284.6 eV from the adventitious carbon. The concentrations of dopants in V_1-x-y_Ti_x_Ru_y_O_2_ thin films were checked by energy dispersive X-ray spectroscopy (EDS). The crystalline structure of the films was examined by X-ray diffraction (XRD) on a Bruke D8 diffractometer (Cu Kα irradiation) and transmission electron microscopy (TEM) on Titan G2 60–300. Raman spectra were characterized by means of a confocal ɑ-Raman spectrometer with the excitation wavelength of 514 nm and an irradiation power of about 0.5 mW (Renishaw inVia). The surface morphology of samples was observed by scanning electron microscopy (SEM, SU8020, Hitachi). The temperature-dependent resistivity of thin films was obtained at a temperature interval of 2 °C according to the thickness and sheet resistance, which was recorded using a four-point probe (SX1934) along with a heating plate.

## Results and Discussion

**Fig. 1:**
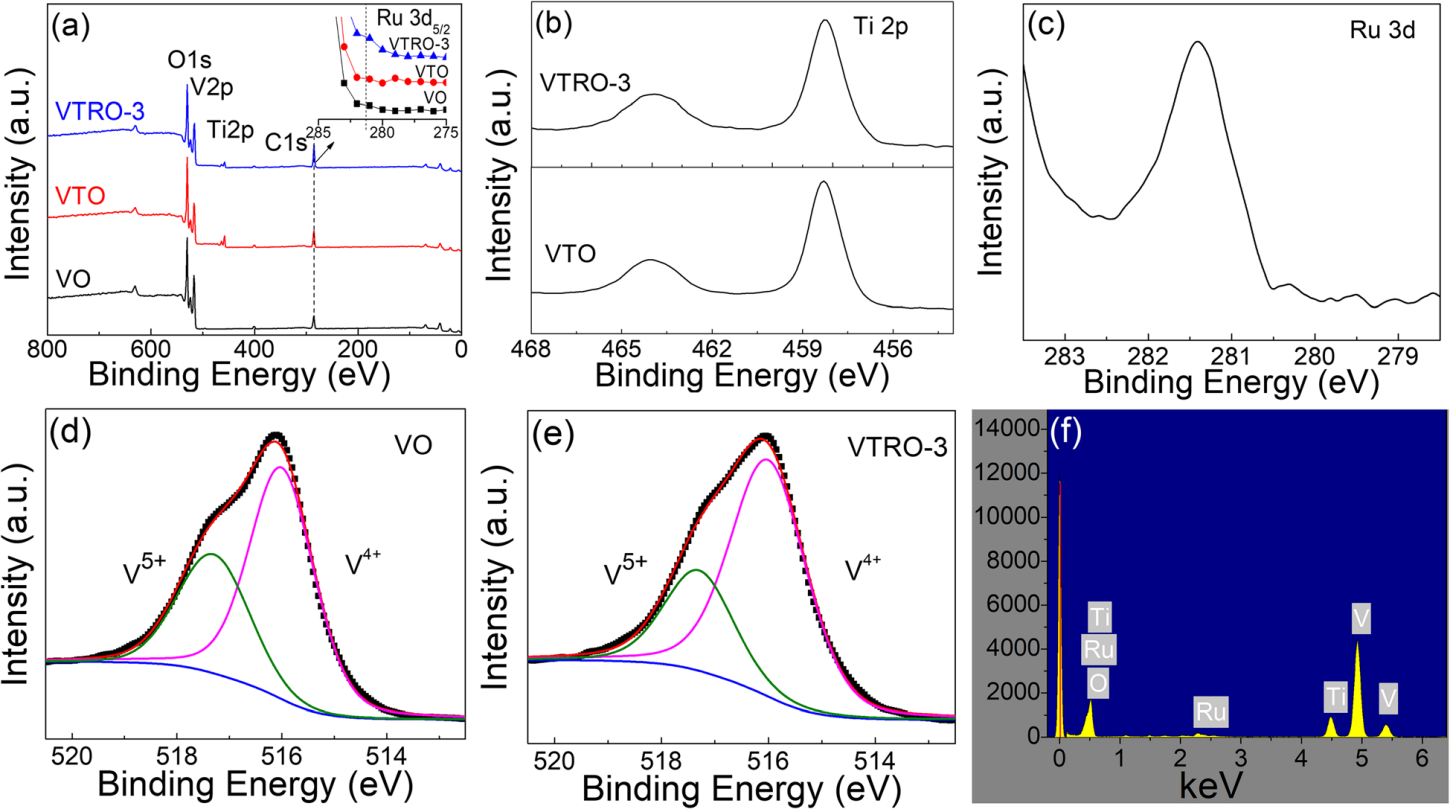
**a** XPS survey spectra of VO, VTO, and VTRO-3, deconvoluted XPS spectra of **b** Ti 2p, and **c** Ru 3d for VTRO-3, **d** V 2p_3/2_ XPS spectra for VO and VTRO-3, **e** EDS spectrum of VTRO-3

The chemical states of dopants in the films were determined by XPS analyses. Figure 1 a shows the XPS survey spectra of VO, VTO, and VTRO-3, clearly showing the strong peaks of V2p, O1s, Ti2p, and C1s. The peak of Ru 3d in V_1-x-y_Ti_x_Ru_y_O_2_ thin films as a shoulder signal of about 281.4 eV can be observed near the C 1 s peak [15]. The successful incorporation of Ti^4+^ and Ru^4+^ ions into the VO_2_ lattice is demonstrated by the Ti 2p peak and the Ru 3d peak of VRTO-3 in Fig. 1 b and c. The Ti 2p_1/2_ peak at 464.0 eV, the Ti 2p_3/2_ peak at 458.3 eV, and splitting energy of 5.7 eV for the Ti 2p doublet indicate the oxidation state of Ti^4+^ ions in VTO and VTRO-3 [16]. Figure 1 c exhibits the Ru 3d XPS spectrum for VTRO-3. The binding energy of 281.4 eV suggests the presence of Ru^4+^ ions in VTRO-3 [16]. The presence of Ti and Ru elements can be further verified by EDS analysis as shown in Fig. 1f. The doping concentrations of Ti and Ru elements (x, y in V_1-x-y_Ti_x_Ru_y_O_2_), obtained by EDS analyses, for all the samples are listed in Table 1. High-concentration Ti was introduced into V_1-x-y_Ti_x_Ru_y_O_2_ thin films. The doping level of Ru in the thin films was well controlled by varying the number of V/Ru alloy pieces.

**Table 1:** Doping levels of Ti and Ru, crystallite size, resistivity, and TCR of all the samples

Sample no.	VO	VTO	VTRO-1	VTRO-2	VTRO-3
Ti concentration (x, %)	–	17.6	17.1	16.7	16.5
Ru concentration (y, %)	–	–	0.65	1.36	1.87
Crystallite size (nm)	25.5	27.6	24.7	17.2	12.5
Resistivity (at 26 °C, Ω·cm)	13.5	12.8	6.53	3.14	1.55
TCR (%/°C)	− 3.13	− 3.46	− 3.54	− 3.46	− 3.47

Moreover, the oxidation states of vanadium ions in films were also analyzed from the deconvoluted V 2p_3/2_ peaks using the Shirley function [17–19]. Figure 1 d and e shows the high-resolution V 2p_3/2_ XPS spectra for VO and VTRO-3. The V 2p spectra both consist of two peaks at 517.4 eV, indicative of V^5+^, and 516.1 eV, indicative of V^4+^ [20]. The appearance of V^5+^ ions could be ascribed to natural oxidation of the sample surface during storage in the air [21, 22]. Specifically, the relative contents of V^5+^ species in VO and VTRO-3, estimated from the integrated intensity of V 2p peak shown in Fig. 1 d and e, are 34.5% and 28.0%, respectively. The relative contents of V^4+^ species in VO and VTRO-3 are 65.5% and 72.0%, respectively. This indicates that V_1-x-y_Ti_x_Ru_y_O_2_ thin film shows higher stability than undoped VO_2_.

**Fig. 2:**
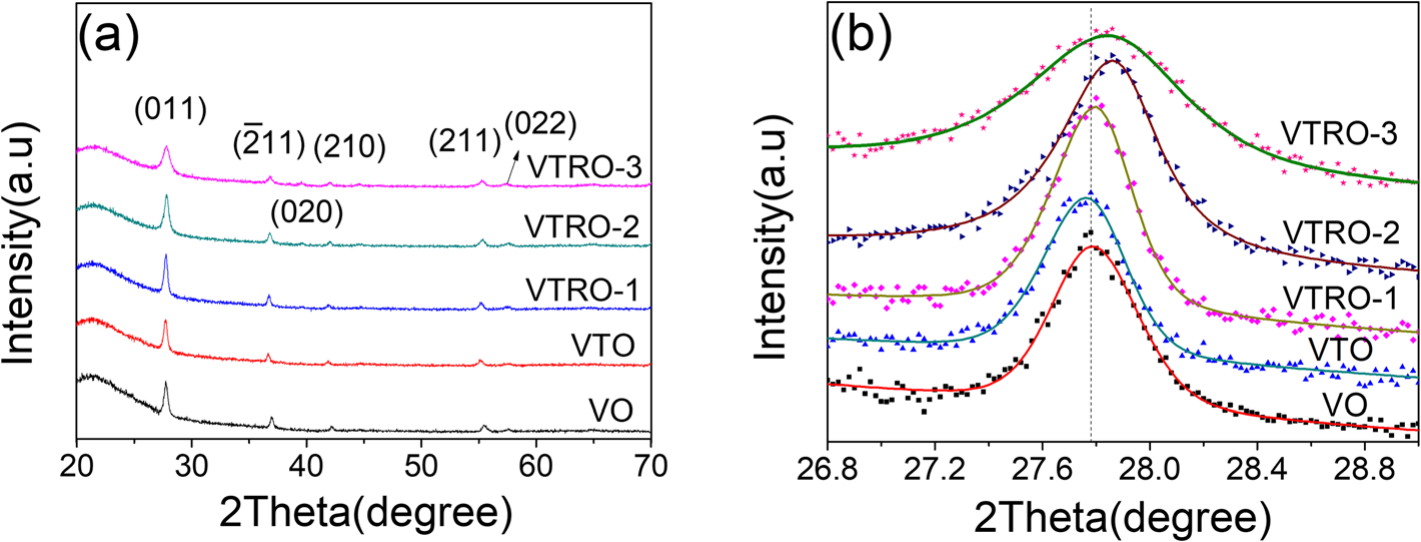
**a** XRD patterns and **b** close-up views of (011) peaks of all the samples

To confirm the crystalline structures, XRD patterns of all the samples were collected (Fig. 2a). All the films exhibit monoclinic structure of VO_2_ (PDF No. 43-1051) [23]. For all the films, the (011) peak seems to be of higher intensity than the other peaks, revealing a preferential growth along (011) facet. No diffraction peaks from other vanadium oxide (V_2_O_3_, V_2_O_5_) [22] or titanium/ruthenium oxide phases can be detected [24]. Also, it is worth noting that V^5+^ ions are probed by XPS while there are no characteristic peaks of the V_2_O_5_ phase in XRD patterns. Considering that XPS is a surface-sensitive technique and the XRD analysis reveals the lattice structure of the whole sample, the presence of V^5+^ ions is believed to be derived from surface oxidation during storage and it exists only on the surface of samples as reported previously [24–27] .

Figure 2 b further shows the close-up views of (011) peak for all the samples after fitting with Lorentzian function. Compared to VO, the (011) diffraction peak of VTO moves from 27.78 to 27.76°. This implies Ti-doping causes a slight increase of the interplanar spacing of (011) facet due to the substitutional presence of Ti in monoclinic VO_2_ [28, 29]. As for V_1-x-y_Ti_x_Ru_y_O_2_, the peak position of the (011) facet shift toward a larger angle (from 27.78° for VO to 27.86° for VTRO-2), indicating that the interplanar lattice spacing varies along (011) facet. This should originate from the replacement of some V^4+^ ions in the monoclinic lattice by Ru^4+^ with a larger ionic radius. According to the Scherrer’s formula, the average crystallite size was estimated from the diffraction data of (011) facet by the Scherrer equation [30]. VTO has larger crystallite size than VO (Table 1). This reveals that Ti-doping promotes the growth of VO_2_ crystallites. But the addition of Ru reduces the crystallite size of films. With increasing the concentration of Ru, V_1-x-y_Ti_x_Ru_y_O_2_ thin films (VTRO-1, VTRO-2, VTRO-3) exhibit gradually reduced crystallite size. Our previous work has demonstrated that Ru^4+^ ions in the VO_2_ lattice inhibit the growth of VO_2_ crystallites in Ru-doped VO_2_ thin films [24]. Similarly, the Ru^4+^ ions suppress the coalescence of adjacent crystallites in V_1-x-y_Ti_x_Ru_y_O_2_ thin films, thus decrease the crystallite size of films.

**Fig. 3:**
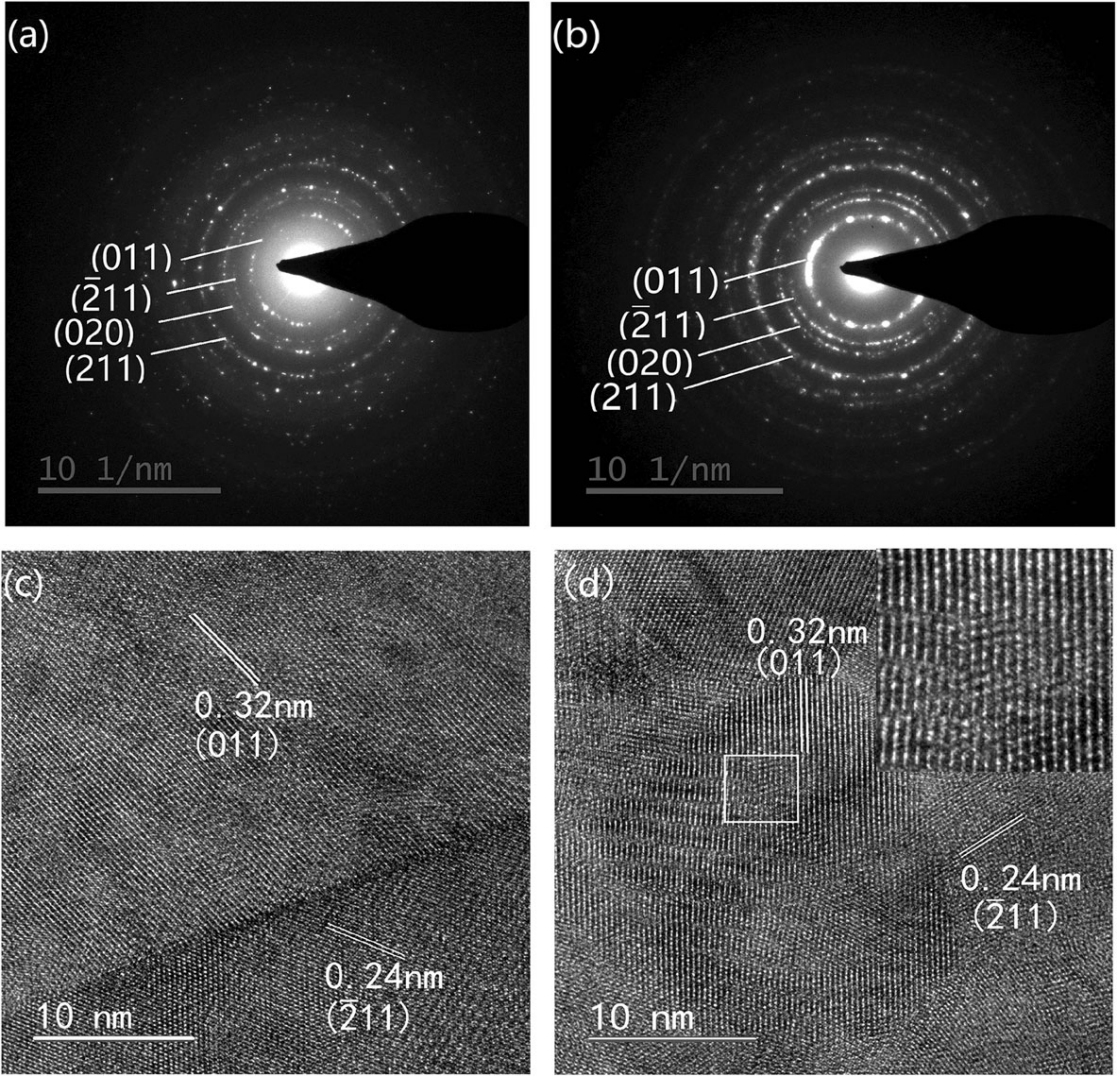
**a** and **b** SAD patterns, **c** and **d** HRTEM images of VO and VTRO-3

The direct observation of the monoclinic lattice in VO and VTRO-3 was performed by means of TEM analysis [31–33]. Figure 3 a and b shows the selective area diffraction (SAD) patterns of VO and VRTO-3. They exhibit clear series of Debye-Scherrer diffraction rings, which can be indexed as monoclinic VO_2_. This suggests the monoclinic polycrystalline feature of undoped VO_2_ and V_1-x-y_Ti_x_Ru_y_O_2_ thin films, which is accordant with the XRD analyses. The high-resolution TEM (HRTEM) images shown in Fig. 3 c and d reveal the clear lattice fringes from monoclinic VO_2_. This further demonstrates that V_1-x-y_Ti_x_Ru_y_O_2_ thin films have the monoclinic structure as the undoped one (VO) [34]. But the insert in Fig. 3d shows the distortion of local lattice fringes in a crystallite of VTRO-3. This indicates that the introduction of Ti and Ru dopants causes obvious disturbance in the lattice of monoclinic VO_2_.

**Fig. 4:**
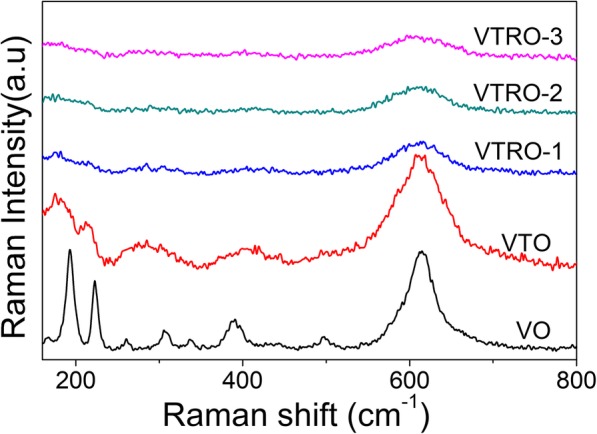
Room-temperature Raman spectra for undoped VO_2_, Ti-doped VO_2_ and V_1-x-y_Ti_x_Ru_y_O_2_ thin films

Figure 4 shows the Raman spectra obtained at RT for the films. All the Raman peaks for VO can be attributed to the A_g_ and B_g_ phonon modes from the monoclinic VO_2_ [35]. No Raman modes from V_2_O_5_ can be observed [24]. Three prominent Raman modes (ω_1_ around 193 cm^−1^, ω_2_ around 223 cm^−1^, and ω_3_ around 613 cm^−1^) are used for further probing the influence of the doping on the crystalline structure of VO_2_ thin films. Ti-doped VO_2_ thin film (VTO) has the similar high-frequency phonon mode (ω_3_) as VO_2_ (VO), typical of monoclinic VO_2_. Differently, two low-frequency modes (ω_1_ and ω_2_) in VTO exhibit obvious redshift compared with undoped VO_2_. The low-frequency modes ω_1_ and ω_2_ can be ascribed to the V-V vibrations [36]. The redshift of ω_1_ and ω_2_ indicates Ti^4+^ ions was introduced into the zigzag V-V chains in monoclinic VO_2_ [37], which decreases the Raman frequencies of the V-V vibrations due to the local structure perturbations around Ti^4+^ ions.

The high-frequency phonon mode ω_3_ is still observed for V_1-x-y_Ti_x_Ru_y_O_2_ thin films, which suggests the presence of monoclinic VO_2_. This is consistent with the XRD and TEM analyses. But their Raman intensities of ω_3_ outstandingly decrease compared with VO and VTO. The other Raman peaks remarkably weaken, even disappear with increasing the Ru concentration. This indicates that there is local disturbance in monoclinic VO_2_ lattice due to the existence of Ti and Ru ions. The previous work has demonstrated that the Ru^4+^ ions in the VO_2_ lattice conduce to inducing the local tetragonal symmetry in the monoclinic framework since the Ru–O coordination exhibits an almost identical symmetry to tetragonal VO_2_ [24, 38]. The tetragonal symmetry has lower Raman activity than the monoclinic phase [39]. Thus, the V_1-x-y_Ti_x_Ru_y_O_2_ thin films show much lower Raman intensity.

**Fig. 5:**
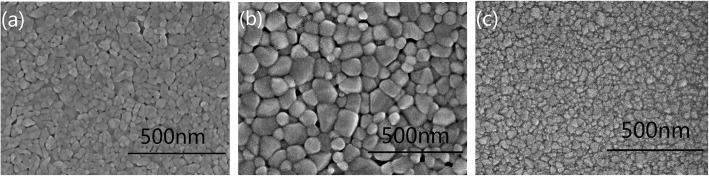
SEM images of the surface morphologies for **a** VO, **b** VTO, and **c** VTRO-3

Figure 5 shows the SEM surface morphologies for VO, VTO, and VTRO-3. The undoped VO_2_ film is mainly composed of particles with size around 50–100 nm (Fig. 5a). Ti-doping obviously influences the surface morphology of VO_2_ films. VTO has a bigger particle size than VO (Fig. 5b). This further indicates that Ti-doping facilitates the growth of VO_2_ crystallites, which is accordant with the XRD data. Differently, VTRO-3 has a denser and smoother surface morphology than VO and VTO (Fig. 5c), which is preferable for fabricating the high-quality pixels in a mircobolometer. Dense surface morphology of VTRO-3 should originate from the inhibition effect of Ru^4+^ ions in VO_2_ lattice on the crystalline growth as revealed by the XRD analysis. Ru^4+^ ions suppress the coalescence of VO_2_ grains by restraining the grain boundary (GB) mobility [24]. VTRO-3 has smaller crystallite size than VO and VTO (Table 1). As a result, smaller grains in VTRO-3 constitute denser films than VO and VTO as shown in Fig. 5.

**Fig. 6:**
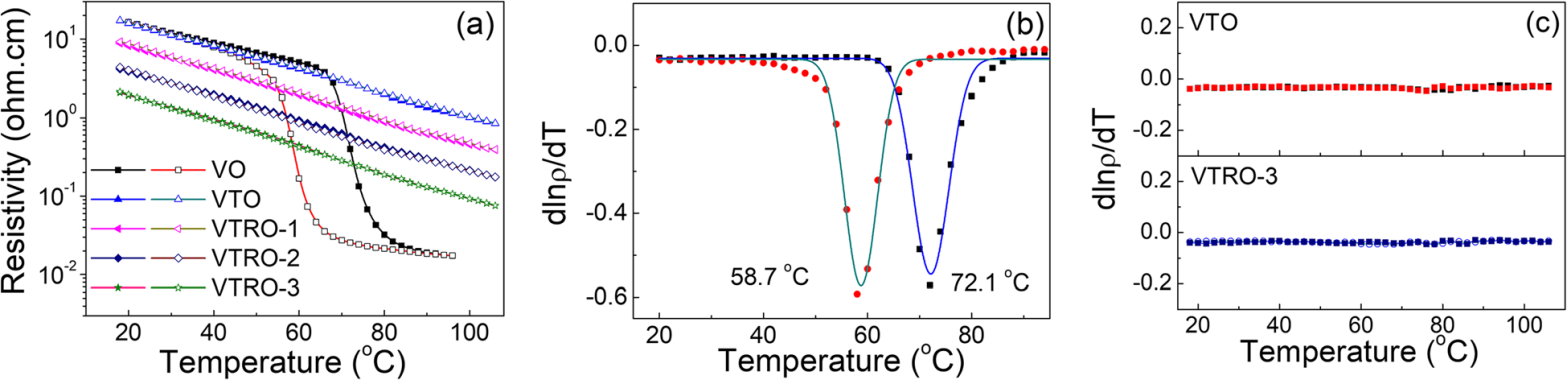
**a** Temperature dependence of ρ for all the samples, plots of dln ρ/dT vs. T for **b** VO and **c** VTO and VTRO-3

Figure 6 a compares the temperature dependence of resistivity (ρ) for undoped VO_2_ film and V_1-x-y_Ti_x_Ru_y_O_2_ thin films. VO has a typical SMT feature of polycrystalline VO_2_ thin films with a SMT amplitude (ratio of the resistivity at 26 °C to the one at 90 °C) of about 3 orders of magnitude, a hysteresis width of 13.4 °C, and the SMT temperature of 72.1 °C (obtained from the plot dln ρ/dT vs. T in Fig. 6b) [40–42]. Interestingly, Ti-doped thin film (VTO) exhibits no abrupt change of resistivity with temperature from RT to 106 °C (Fig. 6c) although it has the same monoclinic structure at RT as VO. This indicates that the SMT of VO_2_ is restrained by Ti-doping with high concentration. The no-SMT feature can avoid the hysteresis and strain changes due to the SMT of VO_2_ across the SMT temperature, which is valuable for the application in microbolometers. With further doping with Ru, the no-SMT feature is maintained in V_1-x-y_Ti_x_Ru_y_O_2_ thin films (Fig. 6c). Moreover, the resistivity of thin films at RT obviously decreases with the increase of Ru concentration (Table 1). The resistivity at RT of VTRO-3 (1.55 Ω·cm) is only one-eighth of VO (13.5 Ω·cm). Generally, the resistivity of polycrystalline films includes grain resistivity and GB resistivity. The decrease of grain size in films results in the increase of GB density, thus increases resistivity owing to GB scattering [43]. VTRO-3 has smaller grain size than VO as revealed by the SEM analysis (Fig. 5). The GB resistivity in VTRO-3 should be larger than that in VO due to increased GB density. But the predicted change trend of GB resistivity with grain size contradicts the change of film resistivity with doping. Therefore, the grain resistivity, rather than GB one, could play a predominant role in the resistivity of VO_2_ polycrystalline thin films. The outstandingly reduced resistivity of VTRO-3 could result from the remarkable decrease of grain resistivity due to the incorporation of Ru^4+^ ions. Substitutional Ru^4+^ ions conduce to induce local tetragonal symmetry in monoclinic VO_2_ lattice, which has been demonstrated by previous work [24]. This causes the upward shift of the maximum of valence band and increase of the density of states of the V 3d electrons, which results in the remarkable decrease of grain resistivity. Thus, VTRO-3 exhibits much lower resistivity than VO. Lower resistivity of thermal sensitive materials generally indicates smaller noise and larger electrical magnification for microbolometer devices, thus higher sensitivity of microbolometers [2]. More importantly, VTRO-3 with low resistivity has large TCR (3.47%/°C), similar to undoped VO_2_ thin film (VO). It is reasonable since semiconductor VO_2_ with monoclinic structure generally exhibits large TCR [44]. As revealed by XRD, Raman, and TEM analyses, V_1-x-y_Ti_x_Ru_y_O_2_ thin films have same monoclinic structure as undoped VO_2_. So, they retain high TCR as monoclinic VO_2_. The TCR value of VTRO-3 is 1.7 times VO_x_ thin films used in commercial microbolometers (about 2%/°C). This is valuable for increasing the sensitivity of microbolometers since it is proportional to the TCR of thermal-sensitive materials [1]. Therefore, V_1-x-y_Ti_x_Ru_y_O_2_ thin film with preferred dopant concentrations (VTRO-3) has attractive characteristics (no-SMT feature, low resistivity, and high TCR) of thermal-sensitive materials for high-performance microbolometers. Furthermore, V_1-x-y_Ti_x_Ru_y_O_2_ thin film exhibits superior trade-off performance to other vanadium oxide-based thermal-sensitive thin films as shown in Table 2. This indicates that V_1-x-y_Ti_x_Ru_y_O_2_ could be a promising thermal-sensitive material for microbolometers.

**Table 2:** TCR, RT resistivity, and processing temperature of V_0.8163_Ti_0.165_Ru_0.0187_O_2_ and other vanadium oxide-based thermal-sensitive thin films previously reported

Material	−TCR (%/°C)	Resistivity (Ω·cm)	Processing temperature (°C)	References
VO_x_	~ 2.7	2	No heating	[45]
Mo-doped VO_x_	4.0–4.5	> 1000	300	3
Mo-doped VO_x_	2.5	0.3	No heating	[45]
Nb-doped VO_x_	2.1	0.5	No heating	[45]
Ti-doped VO_x_	2.5	~ 360	370	[46]
Ta-doped VO_x_	3.47	9.32	400	[47]
V_0.8163_Ti_0.165_Ru_0.0187_O_2_	3.47	1.55	400	This work

**Fig. 7:**
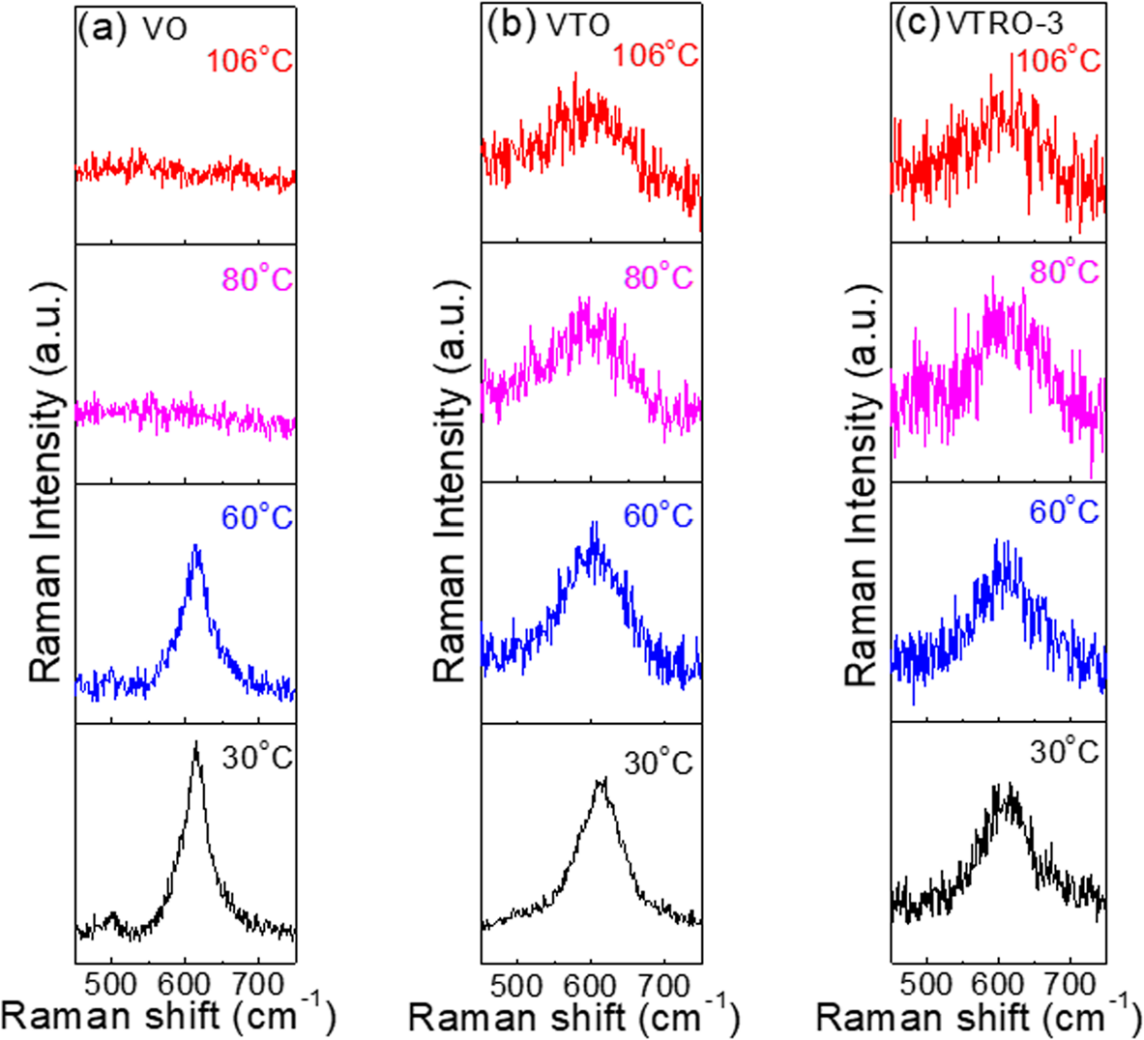
Temperature-dependent Raman scattering characteristics of **a** VO, **b** VTO, and **c** VTRO-3 during the heating

In order to investigate the mechanism resulting in the no-SMT feature in Ti-doped VO_2_ and V_1-x-y_Ti_x_Ru_y_O_2_ thin films, the Raman spectra of VTO and VTRO-3 are acquired at different temperature. As a control, the temperature dependence of the Raman spectrum for undoped VO_2_ thin film (VO) is shown in Fig. 7 as well. Considering that the high-frequency mode ω_3_ is generally reckoned as a fingerprint for the monoclinic VO_2_ [36], the change of this peak with temperature is analyzed. As indicated in Fig. 7a, a clear Raman peak from ω_3_ can be observed for VO before the SMT although the integrated Raman intensity decreases from RT to 60 °C. After the SMT, no Raman peak from ω_3_ can be probed due to the complete structural transition from monoclinic to tetragonal lattice [39]. Differently, the ω_3_ peak can be observed for VTO till 106 °C (Fig. 7b). This indicates the existence of monoclinic VO_2_ in VTO from RT to 106 °C. It has reported that Ti-doping increases the SMT temperature of VO_2_ for a low doping level [48, 49]. But the SMT temperature saturates at 80–85 °C as the doping level reaches above about 8at% [37, 50]. The previous literature demonstrated the SMT amplitude of Ti-doped VO_2_ thin films obviously decreases with Ti-doping level, owing to outstanding increase of the resistivity for the metal state [48]. This could originate from stronger Ti–O bonds than V–O ones. It is well-known that the SMT of VO_2_ is associated with structural transformation from monoclinic phase to tetragonal phase [51]. Compared with the tetragonal phase, monoclinic VO_2_ has remarkably lowered symmetry, which is characterized by zigzag V-V chains with two V-V distances (2.65 and 3.12 Å) [51, 52]. As the temperature rises across the SMT temperature, zigzag V-V chains in the monoclinic phase are transformed into linear V-V chains with a unique V-V distance of about 2.85 Å in the tetragonal phase. Ti has more negative standard heat of formation of oxides than V [53]. This indicates that Ti–O bonds are stabler than V–O bonds. For Ti-doped VO_2_, strong Ti–O bonds stabilize the zigzag V-V chains around them due to the pinning effect. This causes some monoclinic domains to be kept in tetragonal lattice across the SMT. As a result, the post-SMT resistivity of Ti-doped VO_2_ films obviously increases with Ti-doping level since monoclinic VO_2_ has much higher resistivity than tetragonal one. As the concentration of Ti reaches a relatively high value, such as about 17% for VTO, most of monoclinic structures are maintained after the temperature goes above the SMT temperature of VO_2_. As a result, monoclinic structure can be detected in VTO till 106 °C (Fig. 7b). Similar mechanism works for V_1-x-y_Ti_x_Ru_y_O_2_ thin films since Ti^4+^ ions with equivalent concentration to VTO are doped into VTRO thin films. So, the monoclinic structure can be also observed in VTRO-3 till 106 °C as shown in Fig. 7c. Enhanced stability of monoclinic structure causes the no-SMT feature in Ti-doped VO_2_ thin film and V_1-x-y_Ti_x_Ru_y_O_2_ thin films.

**Fig. 8:**
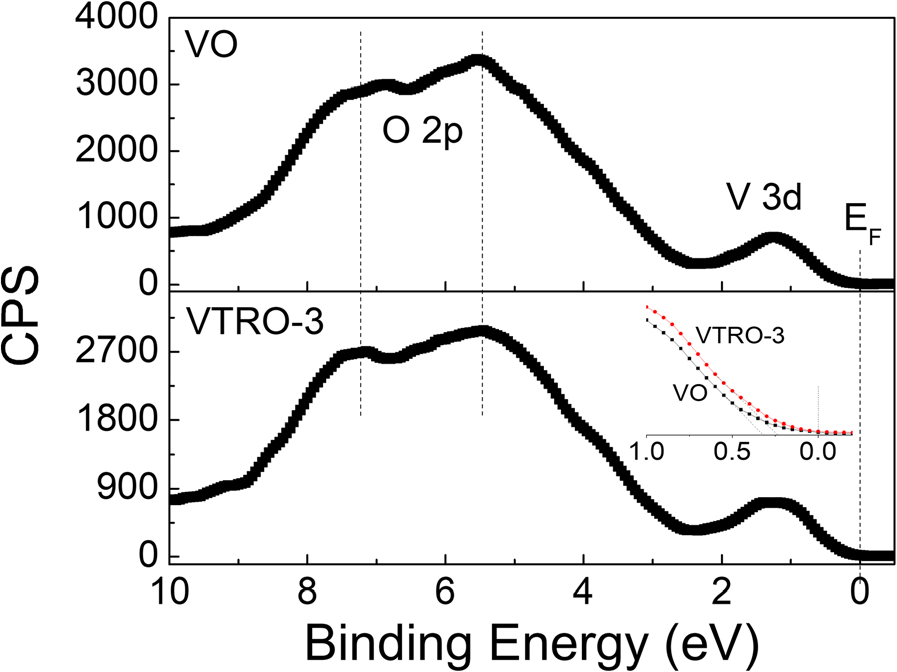
XPS VB spectra of VO and VTRO-3. The inset is the close-up views of VB spectra around E_F_

Low RT resistivity of V_1-x-y_Ti_x_Ru_y_O_2_ thin films should result from the enhanced local symmetry in monoclinic lattice through the substitutional doping of Ru^4+^ ions [24]. Figure 8 shows the XPS valence band (VB) spectra of VO and VTRO-3. Their VB spectra exhibit a two-region structure, consisting of a broad O 2p band and a V 3d band. The band edge at about 0.3 eV reveals the semiconductor state of undoped VO_2_ (VO). Compared with VO, a shift of the V 3d band towards the Fermi level (E_F_) can be observed for VTRO-3. Moreover, the ratio of the integrated intensity of the V 3d band to that of the O 2p band for VTRO-3 (6.23%) is larger than that for VO (4.62%). This suggests that the density of states (DOS) of the V3d band for VTRO-3 increases compared with that for VO [24, 54]. According to the Goodenough’s model, the zigzag V-V chains in monoclinic VO_2_ causes the splitting of the d_||_ band of V 3d electrons into lower and upper d_||_ bands, which results in a bandgap. Thus, monoclinic VO_2_ exhibits a semiconductor state [41, 55]. After doping with Ru^4+^ ions, enhanced local symmetry weakens the splitting of the d_||_ band. This leads to the upward shift of the maximum of VB and the increase of the DOS of the V 3d band [24]. So, more electrons can jump at RT from the VB to the conduction band. Therefore, V_1-x-y_Ti_x_Ru_y_O_2_ thin films have much lower RT resistivity than undoped one.

## Conclusions

V_1-x-y_Ti_x_Ru_y_O_2_ thin films have been prepared by a reactively magnetron co-sputtering process followed by annealing at 400 °C. Ru^4+^ and Ti^4+^ ions are incorporated into VO_2_ monoclinic lattice by substitution. Although V_1-x-y_Ti_x_Ru_y_O_2_ thin films have the same monoclinic structure as undoped VO_2_, the co-existence of Ti and Ru ions deceases the crystallite size of films. This results in smoother surface morphology than VO_2_ thin films. Ti^4+^ ions in the V-V chains of monoclinic VO_2_ stabilize, to some extent, the zigzag V-V chains owing to the pinning effect due to stronger bond strength of Ti–O bonds than V–O bonds. This brings about the no-SMT feature of Ti-doping and Ti-Ru co-doped thin films. V_1-x-y_Ti_x_Ru_y_O_2_ thin films with monoclinic structure exhibit large TCR as monoclinic VO_2_. Enhanced local symmetry due to the Ru-doping leads to much lower RT resistivity for V_1-x-y_Ti_x_Ru_y_O_2_ thin films than undoped one. V_1-x-y_Ti_x_Ru_y_O_2_ is one of promising thermal-sensitive materials for fabricating high-performance small-pixel microbolometers.

## Data Availability

All data and materials are fully available without restriction.

## Abbreviations

SMT: Semiconductor-metal transition
VO_2_: Vanadium dioxide
TCR: Temperature coefficient of resistance
RT: Room temperature
MEMS: Micro-electromechanical system
VO_x_: Vanadium oxide
DC: Direct current
XPS: X-ray photoelectron spectroscopy
BEs: Binding energies
EDS: Energy dispersive X-ray spectroscopy
XRD: X-ray diffraction
TEM: Transmission electron microscopy
SEM: Scanning electron microscopy
SAD: Selective area diffraction
FFT: Fast Fourier transform
